# Correction: Collective motions in the primary coordination sphere: a critical functional framework for catalytic activity of the oxygen-evolving complex of photosystem II

**DOI:** 10.1039/d5sc90135a

**Published:** 2025-06-12

**Authors:** Hiroshi Isobe, Takayoshi Suzuki, Michihiro Suga, Jian-Ren Shen, Kizashi Yamaguchi

**Affiliations:** a Research Institute for Interdisciplinary Science, Okayama University Okayama 700-8530 Japan h-isobe@cc.okayama-u.ac.jp; b Center for Quantum Information and Quantum Biology, Osaka University Toyonaka Osaka 560-0043 Japan

## Abstract

Correction for ‘Collective motions in the primary coordination sphere: a critical functional framework for catalytic activity of the oxygen-evolving complex of photosystem II’ by Hiroshi Isobe *et al.*, *Chem. Sci.*, 2025, https://doi.org/10.1039/d5sc02386f.

The authors regret that a typographical error was introduced into eqn (1) during the editing process. The corrected equation is shown below.1
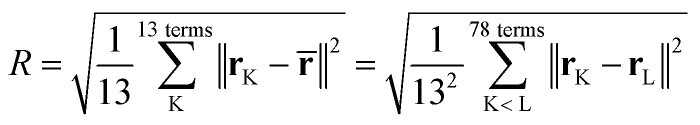


The Royal Society of Chemistry apologises for these errors and any consequent inconvenience to authors and readers.

